# The Early Detection and Case Management of Skin Diseases With an mHealth App (eSkinHealth): Protocol for a Mixed Methods Pilot Study in Côte d’Ivoire

**DOI:** 10.2196/39867

**Published:** 2022-09-21

**Authors:** Rie R Yotsu, Sakiko Itoh, Koffi Aubin Yao, Kouamé Kouadio, Kazuko Ugai, Yao Didier Koffi, Diabate Almamy, Bamba Vagamon, Ronald E Blanton

**Affiliations:** 1 Department of Tropical Medicine Tulane School of Public Health and Tropical Medicine New Orleans, LA United States; 2 School of Tropical Medicine and Global Health Nagasaki University Nagasaki Japan; 3 Department of Dermatology National Center for Global Health and Medicine Shinjuku Japan; 4 Department of Genome Informatics Graduate School of Medicine Osaka University Suita Japan; 5 Hope Commission International Abidjan Cote D'Ivoire; 6 Pasteur Institute Abidjan Cote D'Ivoire; 7 Centre Suisse de Recherches Scientifiques en Côte d’Ivoire Abidjan Cote D'Ivoire; 8 National Buruli Ulcer Control Program Ministry of Health of Côte d’Ivoire Abidjan Cote D'Ivoire; 9 Department of Dermatology Universite Alassane Ouattara Bouaké Cote D'Ivoire; 10 Raoul Follereau Institute Côte d’Ivoire Adzopé Cote D'Ivoire

**Keywords:** skin diseases, neglected diseases, skin NTDs, teledermatology, telemedicine, remote consultation

## Abstract

**Background:**

There is a high prevalence of skin diseases sub-Saharan Africa, including skin neglected tropical diseases (NTDs) that could lead to lifelong disabilities and deformities if not diagnosed and treated early. To achieve early detection and early treatment of these skin diseases, we developed a mobile health app, eSkinHealth.

**Objective:**

This paper outlines a protocol for evaluating the effect of our eSkinHealth app in the early detection and effective management of skin diseases in Côte d’Ivoire.

**Methods:**

A mixed methods pilot trial will be conducted in Côte d’Ivoire and will consist of 3 phases: (1) the development and improvement of the eSkinHealth app, (2) a pilot trial to evaluate the usability of the eSkinHealth app for local medical staff in Côte d’Ivoire, and (3) a pilot trial to evaluate the effectiveness of early detection and case management of targeted skin NTDs (Buruli ulcer, leprosy, yaws, and lymphatic filariasis) with the eSkinHealth app in Côte d’Ivoire. The pilot study will be implemented as a 2-arm trial with local health care providers and patients with skin NTDs over a 3-month follow-up period. The local health care providers will be assigned to an intervention group receiving the eSkinHealth app to be used in their daily practices or a control group. Training will be provided on the use and implementation of the app and the diagnostic pipeline to the intervention group only, while both groups will receive training on skin diseases. Our primary outcome is to evaluate the early detection and effective management of skin diseases using the eSkinHealth app in Côte d’Ivoire by the number of cases diagnosed and managed. Additionally, we will evaluate the eSkinHealth app with validated questionnaires and in-depth interviews. Procedures of our methods have been reviewed and approved by the Institutional Review Board of the Ministry of Health, Côte d’Ivoire and by Tulane University in 2021.

**Results:**

This study was funded in 2021. We started the enrollment of patients in February 2022, and data collection is currently underway. We expect the first results to be submitted for publication in 2023.

**Conclusions:**

Our eSkinHealth app is a field-adapted platform that could provide both direct diagnostic and management assistance to health workers in remote settings. The study will provide evidence for the usability and the effectiveness of the eSkinHealth app to improve the early detection and case management of skin NTDs in Côte d’Ivoire and, furthermore, is expected to contribute to knowledge on mobile health approaches in the control of skin NTDs.

**Trial Registration:**

ClinicalTrials.gov NCT05300399; https://clinicaltrials.gov/ct2/show/study/NCT05300399

**International Registered Report Identifier (IRRID):**

DERR1-10.2196/39867

## Introduction

The prevalence of skin diseases is high in sub-Saharan Africa, particularly in children, and the reported prevalence ranges from 23.3% to as high as 80.4% in this age group [1-7]. These diseases are most often overlooked due to a lack of local specialists and lack of experience among Western specialists looking at darker skin [8]. However, if left untreated, even some of the most common skin diseases could have severe complications (eg, scabies could lead to rheumatic fever and nephropathy, as well as often debilitating physical, social, and mental effects that may deprive one of educational and social opportunities [9,10]). Furthermore, some diseases, including leprosy, Buruli ulcer, lymphatic filariasis, and other skin infections, lead to lifelong disabilities and deformities if not diagnosed and treated early [9,11]. These skin infections that prevail in low- and middle-income countries (LMICs) are members of the skin neglected tropical diseases (NTDs) listed by the World Health Organization and targeted for disease control globally.

Observation of the skin could be very informative. Without undergoing invasive examinations requiring special skills and equipment, many skin diseases could be diagnosed with just sufficient patient history and observation of the skin. This is well suited to field settings in LMICs. Photos of the skin lesions could serve as an alternative to direct observation and, if of sufficiently good quality, could allow for the diagnosis to be made on-site or remotely. Telemedicine for dermatology, or teledermatology, is currently an emerging field taking advantage of this unique feature of skin diseases. A few attempts of teledermatology have been made in sub-Saharan African countries and have shown promising results [12-14]. These previous efforts have faced a number of challenges that we plan to overcome with the proposed work with the following features:

- A field-adapted mobile health (mHealth) app that could provide direct diagnostic and management assistance to health care workers in a remote setting: There is no novel tool to support teledermatology, especially in LMICs where internet accessibility and connection quality is a challenge. It is also of note that photos alone are often not adequate to make a correct diagnosis as outlined previously by Resneck et al [15]. Although clinical photos offer essential information, they need to be accompanied by some clinical information to make a more accurate diagnosis. Furthermore, such a tool needs to be optimized for use in the skin of people of color, given that diagnosis of several skin conditions on skin type IV and darker remains a challenge [16].
- A platform for storage of longitudinal patient records for improved follow-up: We have been conducting active surveillance for skin diseases in Cote d’Ivoire [1]. After providing a diagnosis, follow-up should be performed with the patient, especially as most of the skin diseases are chronic in nature; this is an ethical obligation in medicine. However, there is a lack of skills and expertise in dermatology to pursue this, which is a universal situation in most LMICs [17]. Our field is no exception, and it has been challenging to follow-up with our patients without making repeated field visits, which are often a long distance from city centers [18]. In addition, this lack of capability to follow up with patients is partly due to the lack of a system to document patient records. A platform that stores serial photo documentation of the clinical course could guide health care practitioners, both on-site and remotely, to provide better care.
- A platform for formal collection and automatic organization of clinical and image data of the skin: Currently, teledermatology is mostly done in platforms without any formal framework for the collection or organization of data [19], and there is a need for developing such a platform both for direct patient care and epidemiologic purposes, especially for the organization of clinical photos, which is a cumbersome task if done manually. Patient information management is another challenge in developing a successful teledermatology system, ensuring that patient privacy is fully protected. Nowadays, social networking sites such as WhatsApp and Facebook are sometimes used for teledermatology [20,21], but these informal platforms need to be used with care considering patient privacy. If there is a platform that addresses these gaps, this could further support data analysis and quality control.

In summary, with targeted training, a technology-assisted decision support system, and a telemedicine network, local health care workers could be leveraged to enhance the diagnosis and management of these conditions as well as support health care managers in quality control. If an mHealth app that overcomes these current gaps and weaknesses is developed, this could serve as a breakthrough to managing skin diseases in LMICs.

This project is built upon a previous project for the development of a prototype smartphone/tablet app for skin diseases, which we named eSkinHealth. This app is aimed at on-site and remote diagnosis, monitoring, clinical decision support, and geographic mapping of skin diseases, including skin NTDs adapted for use in LMICs and for skin type IV and darker. Through this project, we will develop a powerful and comprehensive but easy-to-use mHealth app that could be used for the diagnosis and management of all types of skin conditions, especially focused for use in LMICs and for skin type IV and darker.

## Methods

### Study Design

A mixed methods pilot trial will be conducted in Côte d’Ivoire where there is coendemicity with multiple skin NTDs, including Buruli ulcer, leprosy, yaws, scabies, and lymphatic filariasis. The trial will consist of 3 phases: (1) development and improvement of the eSkinHealth app and the platform, (2) pilot trial to evaluate the usability of the eSkinHealth app, and (3) pilot trial to evaluate the effectiveness of early detection and case management of skin diseases with the eSkinHealth app. Phases 2 and 3 of the study will be implemented as a 2-arm trial over a 3-month follow-up.

### Participant Inclusion/Exclusion Criteria

Eligible patients will be defined as those who are clinically suspected of or diagnosed with skin NTDs (Buruli ulcer, leprosy, yaws, scabies, and lymphatic filariasis) or suspected of other clinically diagnosed skin conditions, with fewer than three concomitantly identified skin conditions, and able to consent for themselves. Ineligible patients have more than three skin conditions and reside outside of the target site.

Local health care providers (ie, doctors, nurses, or community health workers/volunteers) must meet all eligibility requirements to enroll in the study. Eligible local health care providers are 18 years or older, working at primary health centers or clinics (PHCs) or within the catchment area of the selected PHCs in Côte d’Ivoire, able to read and speak fluent French, willing to participate in the pilot study for the 3-month study duration and use a provided tablet with the eSkinHealth app if they were assigned in the intervention group, and able to consent for oneself. Ineligible local health care providers will be defined as those who are planning to leave the job at the PHCs within the study period and have difficulty operating mobile devices.

### Recruitment Procedure

We will select 10 to 12 PHCs in health districts with multiple skin NTDs coendemicity as our study site and allocate them equally into intervention and control arms. Targeted patients will include individuals with skin diseases identified through skin surveillance activities and who access the selected PHCs for diagnosis and treatment of their conditions. Local health care providers will be recruited purposively from our study site. A total of 44 local health care providers, including nurses and community health workers, will be selected to participate in the study; the number per PHC will be based on the population of the catchment area of the PHC. All participants will be enrolled formally only after signing the informed consent form.

### Intervention

#### Phase 1: Development and Improvement of the eSkinHealth App and the Platform

**Figure 1:**
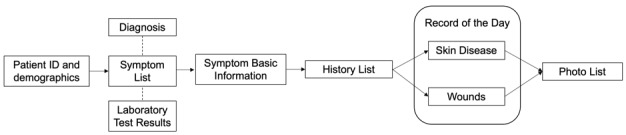
Algorithm in the eSkinHealth app.

**Figure 2:**
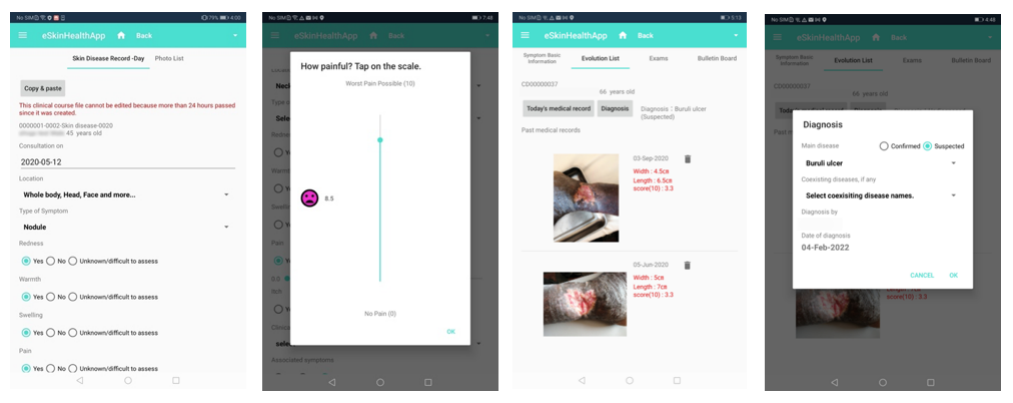
Sample screens of the eSkinHealth app.

The current prototype of the eSkinHealth app is made up of six primary functions, which include (1) patient ID and demographics, (2) symptom list, (3) symptom basic information, (4) history list, (5) record of the day, and (6) photo list (Figure 1). The content of these screens has been developed based on our previous field surveys [1] and from the research team’s experience in managing dermatological patients. We have built-in some special features within each screen (Figure 2). The screen with the patient ID and demographics allows for entry of basic information, automatic calculation of BMI, and assessment of the patients’ nutritional status. The nutritional status could be an important risk factor/indicator for several skin conditions, including wound healing. Patients may present with multiple skin conditions, and this could be organized and managed under the screen of the symptom list. In the screen with the symptom basic information, important information on diagnosing a skin disease is provided as a drop-down choice, so it is not missed and easy to enter. If patients are to return to the clinic, the evolution of their symptom could be seen at a glance in the screen with the history list, allowing comparison with previous symptoms. Lastly, physical examination results of a 1-day visit could be entered in the screen with the record of the day, and clinical photos could be taken, which will be automatically organized in the screen with the photo list. A unique function that we included in our tool is the itchiness and pain visual scale whereby patients themselves could touch the screen and indicate how they are experiencing these sensations each time.

This app is not a disease-specific tool and is fit for use for a wide range of skin conditions with two algorithms: one for nonulcerative skin diseases and another for wounds, a very common manifestation of the skin in LMICs but with different follow-up approaches as compared to other skin conditions. Other special features of the app include patient information security using QR codes. Patients are issued a QR code at their first visit. When they present it to a local health care provider with a device running the eSkinHealth app during follow-up visits, it allows access to their records, making their health records secure and portable.

We will conduct a series of opinion hearings, including expert panel reviews, weekly team meetings, and in-depth interviews and focus group discussions with target end users. In addition, we will develop a web-based platform to manage the case and to have a consultation with a remote health care provider. The app will sync with the platform and will include the following additional functionalities: data overview, search function, and graphical display of data.

#### Phase 2: Pilot Trial to Evaluate the Usability of eSkinHealth

We will conduct a 3-month pilot trial to evaluate the usability of eSkinHealth. Local health care providers in the intervention arm will be provided with a tablet with eSkinHealth installed and a Wi-Fi router, and will be trained on how to use the app by study staff. All local health care providers, irrespective of intervention or control arm, will be provided with a training on the screening and management of important skin diseases. We will then apply a questionnaire survey to investigate the usability of the eSkinHealth app [22]. As for usability, the questionnaire surveys will use the System Usability Scale (SUS) developed and validated by Brooke [23,24]. We will conduct the SUS over a 3-month follow-up with repeated assessments at baseline, the midpoint (6 weeks), and the end of the study (12 weeks). Furthermore, we will conduct several in-depth interviews with:

- Users (ie, local health care providers) to gather feedback on their experience, perceived value, and willingness to use the eSkinHealth app, and identify the obstacles and challenges faced in the implementation of the mobile apps
- Patients (parents or caregivers for children) to assess their willingness to have eSkinHealth used during their consultation (or consultation of their children)
- District officers and program managers to collect and further examine their opinions about the feasibility and advantages and disadvantages of adopting the eSkinHealth app by local health care providers already involved in the delivery of primary health care

#### Phase 3: Pilot Trial to Evaluate the Effectiveness of eSkinHealth for Early Diagnosis and Case Management

The same local health care providers as in phase 2 will participate. They will register patients suspected of the targeted skin NTDs (Buruli ulcer, leprosy, yaws, scabies, and lymphatic filariasis) who provide an informed consent during active case finding and during consultation at PHC throughout the trial period on the eSkinHealth app platform. Clinical data at the time of initial identification and at every follow-up visit until cure will be entered in eSkinHealth, including the photos of their skin lesions. The data will be uploaded when connected to the internet and integrated into one database server. When in need of a consultation, a request will be sent to remote experts/dermatologists in Côte d’Ivoire or members of the global health dermatology community, and advice or a clinical confirmation will be provided. As for the control arm, they will diagnose and manage the cases with skin NTDs as usual following the national standard guidelines. Reporting cases from the control arm will be done by paper-based reports using the consultation registry of the Ministry of Health and the skin NTD reporting forms of the World Health Organization [25].

### Outcome Measurement

For phase 2, we will use a validated questionnaire (SUS). The outcome measurement is an average of the SUS score. Bangor et al [26] found that the SUS was highly reliable (α=.91) and useful over a wide range of interface types [27,28]. The SUS consists of 10 statements with responses in the form of a 5-point Likert scale (eg, 1: strongly disagree; 5: strongly agree). According to Bangor et al [27], a SUS score above 68 would be considered above average. In addition, a SUS score above an 80 is considered excellent and places the product in the top 10% of products tested [29]. We will use the French language version of the SUS. We will assess the usability at baseline, the midpoint (6 weeks), and the end of the study (12 weeks). In-depth interviews will be performed at the end of the study.

For phase 3, differences in case numbers diagnosed and the early detection and follow-up between the intervention group (diagnosis with the app) and the control group (usual diagnosis) will be measured as the primary outcome of this study.

### Data Collection

All survey data will be obtained by study staff. We will use validated and study-specific instruments for data collection in person at enrollment, the midpoint (week 6), and the end point (week 12); the SUS and semistructured interviews will assess the usability of the app and portal. For storage of data, we have been using the Amazon S3 (Simple Storage Service) of the Amazon Web Service server, which offers a safe, secure, highly durable storage infrastructure with continuous backups, regulated under the US Health Insurance Portability and Accountability Act. Only the study team and those registered with the eSkinHealth app system (eg, nurses at primary health care clinics) will have access to data. All paper documentation including the signed consent forms will be stored in a secure cabinet, and access will be available only to the study staffs approved by the institutional review board (IRB).

### Statistical Analysis

#### Sample Size Estimation

As for patients, a convenience sample (N=1320) of participants eligible to participate in this pilot study will be recruited. Sample size calculations were not conducted, as this is a pilot trial. The sample size of 1320 was selected based on the number of participants that could be conveniently recruited and tested within the pilot study time frame. From our previous studies, the average number of patients that got registered to the eSkinHealth app was approximately 10 per PHCs per month.

As for local health care providers, 44 local health care providers (22 local health care providers per group) will be recruited for this study. A power analysis was performed using G*Power 3.1.9.6. An estimated medium effect size of Cohen *f* 0.25 was used to determine the sample size needed for a repeated-measures multivariate analysis of variance (RMANOVA) with three time points and two groups. With 95% power (1 – ß), a sample size of 44 local health care providers is needed to detect the hypothesized medium-sized effects on the various outcomes. This RMANOVA would uncover all large- and medium-sized effects but no small-sized effects.

#### Data Analysis

Statistical analyses will be performed using Stata software (version 16, StataCorp). The threshold for statistical analyses will be set at *P*<.05 in a 2-tailed test. We will summarize the baseline data by group assignment using descriptive statistics: means and SDs will be used for continuous data with normal distribution, medians and IQRs for skewed data, and percentages for categorical data. Our primary outcome, number of diagnosed and early detection will be assessed by mix model analysis of variance, with intervention assignment as a between-group factor and time as a within-subject factor. As for continuous data, we will compare the data between control and intervention group using the *t* test or the Wilcoxon Mann-Whitney test. For categorical data, we will compare the data using the chi-square test.

### Ethics Approval

Procedures of our methods have been reviewed and approved by the IRB of the Ministry of Health, Côte d’Ivoire (No. IRB000111917) and by the Tulane University (IRB 2020-2054-SPHTM). This study is registered at ClinicalTrials.gov (NCT05300399).

## Results

This study was funded in 2021. We started the enrollment of patients in February 2022, and data collection is currently underway. Data collection is expected to be completed in October 2022. We expect the first results to be submitted for publication in 2023.

## Discussion

This paper outlines the protocol for a 3-month pilot trial for evaluating the effect of the eSkinHealth app for the early detection and management of skin NTDs. This app has been our invention, and to our knowledge, there is no other mHealth app of this kind that is developed for the collection of the clinical data of skin diseases in which it can be used both online and offline. It is also portable, and therefore, it can be used in remote communities in LMICs where skin NTDs are endemic and where infrastructure is usually very poor. In terms of teledermatology, while past attempts in using WhatsApp and other social media platforms have shown successful results to an extent [20,21], they do not offer patient health information security. Furthermore, information is exchanged on a one-time basis, and it does not offer a platform for the follow-up of patients. Our system attempts to overcome these challenges.

The outcomes of this study will be primarily assessed by the number of cases of skin NTDs diagnosed and managed using the eSkinHealth app in Côte d’Ivoire, consistent with previous studies in LMICs [30-34]. We will further evaluate it with the SUS score and in-depth interviews. We expect that the average SUS score will be above 68, and the usability of the eSkinHealth app would be considered above average. We hypothesize that the use of the eSkinHealth app will improve the early detection and management of skin NTDs.

Our study may have several limitations. First, although the app can be used offline, an internet connection is required for certain functions. Poor internet connectivity may affect our results. While we can exclude PHCs with known poor internet connectivity, this will form one of the results of our study. We therefore did not include this as our criteria in the selection of the users. Second, the SUS will be evaluated at enrollment, the midpoint (week 6), and the end point (week 12). It is unclear what the effects of the intervention would be if it were prolonged. Lastly, this is a pilot trial involving 10 to 12 PHCs in one or two health districts, and therefore, the study findings may not be generalizable to all health districts in Côte d’Ivoire. These limitations notwithstanding, this study is the first to examine the usability and the effectiveness of the eSkinHealth app to improve the early detection and case management of skin NTDs in Côte d’Ivoire.

The study will provide robust evidence of the usability and the effectiveness of the eSkinHealth app to improve the early detection and case management of skin NTDs in Côte d’Ivoire. Furthermore, given the importance of improving the early detection and case management of skin NTDs in LMICs, these results will provide a compelling rationale for infectious disease policy and decision makers regarding mHealth interventions for skin NTDs in LMICs.

## Appendix

Multimedia Appendix 1 Peer review report from the Center for Scientific Review Special Emphasis Panel - Mobile Health: Technology and Outcomes in Low and Middle Income Countries (National Institutes of Health, USA).

## Abbreviations

IRB: institutional review board
LMICs: low- and middle-income countries
mHealth: mobile health
NTD: neglected tropical disease
PHC: primary health center or clinic
RMANOVA: repeated-measures multivariate analysis of variance
S3: Simple Storage Service
SUS: System Usability Scale

## Data Availability

The data sets generated or analyzed during this study are available from the corresponding author on reasonable request.
