# Supplementary material for: The Early Detection and Case Management of Skin Diseases With an mHealth App (eSkinHealth): Protocol for a Mixed Methods Pilot Study in Côte d’Ivoire
Source: JMIR Res Protoc. 2022 Sep 21;11(9):e39867. doi: 10.2196/39867 (PMC9536527; doi:10.2196/39867)
Supplement: Multimedia Appendix 1 [file resprot_v11i9e39867_app1.pdf]

SUMMARY STATEMENT
(Privileged Communication)

PROGRAM CONTACT:
Brad Newsome
301-827-8170
brad.newsome@nih.gov

Release Date: **04/08/2021**
Revised Date:

Application Number: **1 R21 TW011860-01**

Principal Investigator

YOTSU, RIE

Applicant Organization: TULANE UNIVERSITY OF LOUISIANA

Review Group: **ZRG1 IMST-U (55)**
Center for Scientific Review Special Emphasis Panel
PAR-19-376:Mobile Health: Technology and Outcomes in Low and Middle Income Countries

Meeting Date: **03/24/2021**
Council: **MAY 2021**
Requested Start: **07/01/2021**
RFA/PA: **PAR19-376**
PCC: **MHEALTH**
Dual IC(s): **EB, HD**

Project Title: **MIND-the-SKIN Project: Multi-functional Innovative Digital Toolkit for the Skin Diseases in LMICs and Beyond**
SRG Action: **Impact Score:30 Percentile:15 #**
Next Steps: **Visit https://grants.nih.gov/grants/next_steps.htm**
Human Subjects: **48-At time of award, restrictions will apply**
Animal Subjects: **10-No live vertebrate animals involved for competing appl.**
Gender: **1A-Both genders, scientifically acceptable**
Minority: **5A-Only foreign subjects, scientifically acceptable**
Age: **1A-Children, Adults, Older Adults, scientifically acceptable**

Project Year	Direct Costs Requested	Estimated Total Cost
1	122,871	188,679
2	124,714	191,510
3	192,987	296,349
4	197,134	302,717
5	196,251	301,361
TOTAL	833,957	1,280,616

ADMINISTRATIVE NOTE

1R21TW011860-01 Yotsu, Rie

PROTECTION OF HUMAN SUBJECTS CONCERN

SCIENTIFIC REVIEW ADMINISTRATOR'S NOTE

RESUME AND SUMMARY OF DISCUSSION: It is planned to develop a mHealth app relevant to skin diseases in the Ivory Coast. The R21 phase is to optimize an existing smartphone/tablet app for photographic analysis of skin lesions (that would be especially suitable for dark-skinned people) and to develop a wound-scoring system, followed by an R33 project to evaluate the performance of the app in surveillance and management of neglected tropical diseases that would facilitate wound management. This an important effort – skin diseases in Africa are under-diagnosed, and standardizing photographic analysis of lesions on dark skin would be quite valuable. The investigative team is excellent, and innovation is high. The approach and environment are quite strong. The panel also identified some weaknesses. There is lack of discussion of follow-up in terms of clinical care when serious problems are discovered. The endpoint of the training program needs better explication. There are concerns about consistency of internet connectivity, and about data security. On balance, while considering all types of wounds may be too ambitions, the photos will be important to have and the impact of the work should be substantial.

DESCRIPTION (provided by applicant): The prevalence of skin diseases is extremely high in sub-Saharan Africa, particularly in children. If left untreated, some have debilitating lifelong physical disabilities and deformities, as well as social and mental effects. Among them are skin infections which are listed as members of the Neglected Tropical Diseases (NTDs) by the World Health Organization and targeted for disease control globally. Many of the NTDs, including leprosy, Buruli ulcers, yaws, and lymphatic filariasis, are co-endemic in West Africa. Early detection and treatment are currently the most effective measures for their disease control. However, this is hampered by a paucity of skilled healthcare workers in the remote areas of the LMICs where they prevail. On the other hand, diagnosis of these conditions can be done with patient history and simple skin examination, without undergoing invasive examinations requiring special skills and equipment. This is very well suited to field settings in LMICs. With a targeted training, technology-assisted decision-support system and a telemedicine network, local healthcare workers could be leveraged to enhance the diagnosis and management of the skin conditions. This project proposes to do so through development and validation of an mHealth tool for skin diseases in LMICs: the 'eSkinHealth' application. It is a portable smartphone or tablet application that could provide: a) direct diagnostic and management assistance to healthcare workers in remote setting, and through teledermatology, b) storage of longitudinal patients records for improved follow-up, and c) organization of clinical and image data of the skin. There is a general lack of good clinical photos of skin diseases on dark skin, and collection of photos made through this project could be further used for development of photo guides for wide use and future studies on dermatology of skin of color. The purpose of the proposed research is to complete development and optimization of eSkinHealth and carry out its effectiveness studies on the field. The R21 phase of the work will assess the feasibility, usability, and acceptability of the prototype, validation of a novel built-in wound scoring system, and standardization and optimization of dermatologic photography on dark skin. The R33 phase will evaluate the performance of the app on outcomes of surveillance and management of NTDs and further optimization of photo analysis of skin lesions on dark skin using the image optimization technology. The research will be carried out in a western-African country of Côte d'Ivoire. It will build on our previous work in surveillance of NTDs in rural Côte d'Ivoire and trainings to local healthcare workers, as well as our expertise in mHealth. It will leverage our multi-year collaboration with investigators from Côte d'Ivoire, application developers in Japan, and tropical medicine expertise from Tulane University. If successful, eSkinHealth will be instrumental in overcoming the current gaps and

weaknesses in dermatological services in LMICs providing a breakthrough to management of skin diseases in this underserved populations, as well as contributing to the dermatology of skin of color worldwide, envisioning Skin Health for All.

PUBLIC HEALTH RELEVANCE: While the prevalence of skin diseases are very high in LMICs, especially in children, dermatological services in LMICs are lacking mainly due to lack of clinical skills, and further, ways to document and store clinical course which is important for individual patient follow-up do not exist. If there is a field-adapted platform for data collection and sharing for skin conditions allowing clinical decision support for healthcare providers onsite, and remotely through teledermatology, this could serve as a breakthrough to management of skin diseases in this underserved populations. In this project, we will complete the development and optimization of a powerful and comprehensive, but easy-to-use smartphone / tablet application tool that addresses these gaps, envisioning Skin Health for All.

CRITIQUE 1

Significance: 4
Investigator(s): 2
Innovation: 2
Approach: 4
Environment: 2

Overall Impact: The aim of this project is to build a telehealth program to diagnose skin conditions in Côte d' Ivorie. Generally, the application is well written and organized and the roles of each participant are clear. Aim 1 is to develop an mHealth platform to assist with diagnosis and management of skin lesions with an emphasis on dark skin. The next Aim is to create a medical record for longitudinal storage of records to provide better follow up. The third Aim is to formalize the platform for derm telehealth. The innovative idea to gain information about skin disorders in dark skinned people is important. There is some concern about significance because the proposal covers general skin diseases rather than targeting specific areas of concern. In addition, it is unclear if simply providing diagnosis will decrease mortality. There is also not a clear plan for sustainability, particularly in the treatment realm, after the grant is over. The first Aim of the R33 phase is very well described and has measurable endpoints, but the other R33 Aims do not.

1. Significance

Strengths

- Skin diseases have a high prevalence in Africa and are often underdiagnosed.
- Left untreated, skin disease can lead to morbidity and mortality.
- A telehealth platform for skin health could help remote workers diagnose and manage skin conditions and keep longitudinal records about skin conditions.
- It is intended to collect high-quality photos for a future AI tool.
- The plan to standardize photo analysis of skin lesions on darker skin is an excellent, important Aim, but it is also so broad it that it may be hard to draw conclusions.

Weaknesses

- The research plan Is very broad in terms of skin diseases. It is hard to see a substantial impact if the goal is to diagnose any type of skin disorder.
- There is only brief mention of linkage from diagnosis to treatment. How will this app be used to really make a difference?
- The application would be stronger if it provided more information on the benefit of longitudinal photo capture.
- Remote consultation will be very difficult in areas without internet connectivity.
- It is unclear if simply providing a diagnosis would decrease the prevalence of dermatologic conditions since availability of supplies and cost of treatment is also a factor.

2. Investigator(s)

Strengths

- Dr. Yotsu is an Assistant Professor at Tulane - MD from Japan (dermatology) and PhD in International Health with training in tropical medicine.
- Co-investigator Dr. Blanton is a physician scientist with expertise in epidemiology and international disease; he holds a current RO1 focusing on schistosomiasis.
- Dr. Vagamon is a Dermatology Professor in Côte d'Ivorie where the research will be conducted.
- Dr. Koffi is also a medical doctor in Côte d'Ivorie working on neglected tropical diseases (NTDs).
- Dr. Aubin is a health economist living and working in Côte d'Ivorie who works on study implementation, a cost-effective analysis for this study, and will liaison with the Ministry of Health (MOH) which helps ensure sustainability.
- Dr. Itoh (Ota) has a doctorate in nursing and specializes in bioinformatics. She has been working with the Principal Investigator (PI) in Côte d'Ivorie. Mr. Takagi is the CEO of the software company which will work to develop the app.

Weaknesses

- Dr. Yotsu has limited publications but several are in high impact journals (PLoS) and focus on skin disease in Côte d'Ivoire. She has several international grants but no history of NIH or other US federal funding.
- The team could likely benefit from a health economist if cost effectiveness analysis would be performed.

3. Innovation

Strengths

- There are gaps in knowledge about dermatology in dark skin and this project helps fill that gap.
- The program builds upon work from previous grants that developed an "eSkinHealth" app to diagnose, monitor and provide clinical decision support in LMICs.

Weaknesses

- The project would develop a novel scoring system for skin wounds but it unclear how creating a record of progression over time would impact morbidity and mortality from disease.

4. Approach

Strengths

- Regarding Aim 1, the app is already built and this project provides its clinical validation.
- The first Aim will improve the app based on user input obtained through in-depth interviews.
- Optimizing health care worker (HCW) photos will help in creating a machine learning tool in the future to assist with diagnosis.
- The endpoint of 70% of trained personnel using the app at month 23 is measurable and is a good measure of feasibility.
- The final Aim is to develop a training package that would be used in the larger study.
- The R33 is focused on Buruli ulcer, leprosy, yaws and lymphatic filariasis, which is logical based on experience of the team and potential for impact.
- The qualitative impact evaluation is well thought out and described.

Weaknesses

- The Aim dealing with accurate wound scoring needs to have more detail about specific interventions that would change depending on what the wound looks like on follow-up. What measures would be provided? Are these practical in an LMIC? Are there any data that this is the case?
- There is not much information given on the 32 trainees and how generalizable this data would be for future scale up and sustainability.
- The endpoint of 100% high quality photos is not adequate for a successful training program.
- The proposal states that dermatologists from Kings College of London will serve in a voluntary capacity for remote diagnosis. The letter of support indicates overall support of the project but should specifically clarify their willingness to participate.
- The wound app Aim evaluation is weak - it is not powered and does not include cost-effectiveness analysis language although it mentions this will be done. There are also no measurable endpoints. This Aim is not crucial to the overall application and it may be considered a separate project and could be omitted.
- The image optimization would benefit from some deliverables that could make it easy to use for personnel who did not participate in the formal training. It also lacks measurable endpoints.

5. Environment

Strengths

- Tulane has an excellent global health program and record of successful research.
- The National Buruli Ulcer control program is part of the Ministry of Health and has a history of international collaboration and support from WHO.
- Hope Commission International is an NGO that has headquarters in the US.
- The three sites in Côte d'Ivoire are in close proximity and can easily collaborate.
- The PI has a long history of collaboration with the Japanese partners.

Weaknesses

- No comment provided by reviewer.

Milestones

Strengths

- The milestones listed are reasonable.

Weaknesses

- There is no timepoint given for IRB submission and approval, which could upend the project.
- There is not a plan for scale up and sustainability (particularly in terms of supplies) when the project is complete.

Study Timeline (Specific to applications involving clinical trials)

Strengths

- No comment provided by reviewer.

Weaknesses

- Timeline is ambitious. There are many goals and a very large number of subjects that may not be feasible in this time period.

Protections for Human Subjects:

Acceptable Risks and/or Adequate Protections

- Acceptable plan in place

Data and Safety Monitoring Plan (Applicable for Clinical Trials Only):

Acceptable

- Acceptable plan in place

Inclusion Plans:

- Sex/Gender: Distribution justified scientifically
- Race/Ethnicity: Distribution justified scientifically
- For NIH-Defined Phase III trials, Plans for valid design and analysis:
- Inclusion/Exclusion Based on Age: Distribution justified scientifically
- Plan will include Africans, which is consistent with race distribution

Vertebrate Animals:

Not Applicable (No Vertebrate Animals)

Biohazards:

Not Applicable (No Biohazards)

Applications from Foreign Organizations:
Justified

Select Agents:
Not Applicable (No Select Agents)

Resource Sharing Plans:
Acceptable

- Plan in place

Authentication of Key Biological and/or Chemical Resources:
Not Applicable (No Relevant Resources)

Budget and Period of Support:
Recommended budget modifications or possible overlap identified:

- The budget goes all to Tulane. There are fees to cover the work of the local doctors, field workers, medications and IRB. The request is appropriate but there is a minor concern that there is no subcontract with the involved LMIC.

CRITIQUE 2

Significance: 3
Investigator(s): 3
Innovation: 4
Approach: 4
Environment: 4

Overall Impact: The team proposes to use an existing mHealth platform (eSkinHealth) to provide 1) diagnostic aid and telemedical diagnosis of skin disease in Africa, 2) electronic storage of images, 3) organization of clinical/skin image findings. The approach is thoughtful with clear success criteria for the R21 to R33 transition and implementation science-based evaluation. Innovation is primarily in the implementation of technology, rather than the technology itself. More detail for who the precise user of the mHealth device is, and how this integrates into the existing primary health care infrastructure, would be helpful.

1. Significance

Strengths

- Pediatric skin disease is an important problem in sub-Saharan Africa.
- There are no telederm platforms adapted for use in LMICs.
- Maintaining photo/clinic records is a challenge for care delivery.

- App is currently in preliminary testing in country.

Weaknesses

- No comment provided by reviewer.

2. Investigator(s)

Strengths

- Team has relevant experience.

Weaknesses

- No comment provided by reviewer.

3. Innovation

Strengths

- This mHealth approach could be useful for its intended purpose.
- Focusing on app performance in darker skin pigmentation is an important innovation for global impact.
- Innovation is not primarily in the technology (combining telemedicine + electronic health records) but in the implementation. Successful demonstration could generalize to other specialties.

Weaknesses

- How does this integrate with other medical care needs in the LMIC setting (e.g., acute diarrheal illness, malaria, etc.)?

4. Approach

Strengths

- Aim 1 – thoughtful user-centered approach to evaluating usability
- Aim 2 – Delphi approach to developing wound scoring system
- R33 evaluation could be quite impactful.
- Well described implementation science approach

Weaknesses

- Aim 1 – measurement of usability is not clear prior to the milestones section. What does >68 mean system usability mean? How does it relate to "effectiveness, efficiency, and overall ease of use"?
- It is not clearly described how the app improves diagnosis of Buruli ulcer (or any diagnosis).
- QR code-based patient ID relies on patients not losing the QR code. Is there a backup?
- Vague details on how the tele-dermatology would work in practice.

5. Environment

Strengths

- Team has adequate experience.
- High probability of ongoing work if successful.

Weaknesses

- No comment provided by reviewer.

Milestones

Strengths

- Clear milestones with quantifiable criteria for success of R21.

Weaknesses

- Need to better define exactly how the usability metric is calculated in Aim 1.
- 100% high image quality is a high bar – is this realistic?

Study Timeline (Specific to applications involving clinical trials)

Strengths

- Timeline makes sense.

Weaknesses

- No comment provided by reviewer.

Protections for Human Subjects:

Acceptable Risks and/or Adequate Protections

Data and Safety Monitoring Plan (Applicable for Clinical Trials Only):

Acceptable

Inclusion Plans:

- Sex/Gender: Distribution justified scientifically
- Race/Ethnicity: Distribution justified scientifically
- For NIH-Defined Phase III trials, Plans for valid design and analysis: Not applicable
- Inclusion/Exclusion Based on Age: Distribution justified scientifically

Vertebrate Animals:

Not Applicable (No Vertebrate Animals)

Biohazards:

Not Applicable (No Biohazards)

Applications from Foreign Organizations:

Justified

Select Agents:
Not Applicable (No Select Agents)

Resource Sharing Plans:
Acceptable

Authentication of Key Biological and/or Chemical Resources:
Not Applicable (No Relevant Resources)

Budget and Period of Support:
Recommend as requested

CRITIQUE 3

Significance: 2
Investigator(s): 1
Innovation: 3
Approach: 4
Environment: 3

Overall Impact: This is an initial application to use an app called eSkinHealth, a smartphone or tablet app that logs and provides longitudinal follow up around the development of ulcerative skin disease in the Ivory Coast. The investigators focus on Buruli ulcers, which may cause significant morbidity and is classified as a neglected tropical disease. Part of the issue is that there is high prevalence of disease in sub-Saharan Africa yet understanding of diagnosis and follow up is hampered by the lack of dermatologists who manage this disease, and supporting tools that permit longitudinal follow up for patients suffering from Buruli ulcers. In this proposal, the investigators will use the eSkinHealth app that was previously developed and piloted and adapt it through structured user feedback from key stakeholders. The R21 phase will also develop best practice guidelines and a scoring system for wound assessment and finally develop a photo system to store images of wounds for teaching purposes as well as follow up. The milestones governing the transition to the R33 phase are well defined. The R33 phase itself is rather ambitious; two clinical trials are planned in eight randomized health care regions. If successful, this proposal will have high impact on the management and infrastructure around ulcerative skin disease and wounds in the Ivory Coast. The project team is very strong having had collaborations and involvement of investigators and the Ministry of Health in the Ivory Coast. The study site is also ideal to investigate the use of the app given the high prevalence of Buruli ulcers in this region. There are minor weaknesses around the ability to provide true teledermatology services post study, and the sustainability of the app developed. There are also several concerns in terms of human subjects about data safety and consent, especially of children who may be enrolled in the study. Despite these issues, the research methods overall are sound, and impact is likely to be moderate to high.

1. Significance:

Strengths

- The prevalence of skin disease in sub-Saharan Africa is high, and there is a lack of specialists to help diagnose these diseases, which lead to significant morbidity.
- Diagnosis of skin disease can be supported through low intensity use of mHealth applications to take photos of lesions and telemedicine to foster treatment guidance.
- Understanding the epidemiology of skin disease in Africa is difficult given the lack of infrastructure to support image storage and longitudinal follow up; this proposal seeks to address these important barriers.

Weaknesses

- With increased teledermatology and mHealth tools to diagnose skin disease, especially Buruli ulcers, it is unclear if there is capacity to conduct the intensive months-long wound care that may be required in these cases.

2. Investigator(s):

Strengths

- The PI has expertise in diagnosis of tropic skin diseases and has close collaborators in the Ivory Coast, which will be the prime site for research activities.
- Study investigators in the Ivory Coast have developed clinical infrastructure for managing skin related NTDs. Additionally, there is development of mHealth infrastructure in the Ivory Coast to enable outreach to manage NTDs and connect with international experts.

Weaknesses

- No comment provided by reviewer.

3. Innovation:

Strengths

- Deployment of a tablet-based teledermatology system in sub-Saharan Africa is innovative and seeks to address a fundamental challenge in the diagnosis and treatment of skin disease.
- Obtaining photos of dark-skinned individuals may help advance teaching surrounding skin disease in Africa. This strategy, if successful, will enhance the mHealth capacity of the Ivory Coast.
- Longitudinal assessment of skin disease through an app that can be used online and offline as a dermatology electronic health record is innovative.
- Access to the app by patients is by a QR code given to patients - they can then have a provider scan this QR code and obtain access to longitudinal wound images.

Weaknesses

- Unclear how this app would facilitate linkage to dermatology care.

4. Approach:

Strengths

- Approach in the R21 phase is built around an app that has been developed previously. Several algorithms exist to guide clinicians through management of wounds and other skin diseases.

- R21 development phase uses the modified information systems framework to improve the existing app, integrating user feedback from physicians, nurses, health workers and patients.
- The R33 phase is grounded in the realist evaluative approach and seeks to understand the effects of screening, referral and teledermatology to manage specific ulcerative skin disease.

Weaknesses

- The R21 phase assesses the usability of the app in a 3-month trial period, but without specific skin conditions, it is unclear how often the app would be used by different stakeholders.
- Unclear how many in depth interviews are conducted in the R21 phase.
- The applicants report that dermatologists from King's College London and International Foundation for Dermatology will support the teledermatology component of this app, but it is unclear how the dermatologists will be recruited and the number of dermatologists needed to sustain the teledermatology capabilities to support community health worker (CHW) screening patients in the Ivory Coast.
- The goals of the R33 phase are ambitious - to conduct two separate clinical trials during the three years of the R33. It is unclear if patients are recruited out of the same clinics as the Aim 1 clinical trial (R33 phase), or whether there would be bias for CHWs who have used the app in one study but may not be using it in the second.

5. Environment:

Strengths

- Strong facilities at Tulane and especially at the IRFCI and other participating Cote d'Ivorie sites suggest an adequate institutional environment to conduct the proposed work.
- On site facilities in the Ivory Coast have the infrastructure required to conduct clinical trials.

Weaknesses

- No indication of strategies to conduct outreach in communities within the Ivory Coast.

Milestones:

Strengths

- There are strong and detailed milestones for transition to R33 phase. Quantified measures with benchmarks for passage to the R33 are well defined.
- Milestones detail long term impact of modules developed in the R21 phase that will be used both in the R33 phase and can be applied outside of the context of the grant.

Weaknesses

- No comment provided by reviewer.

Study Timeline (Specific to applications involving clinical trials):

Strengths

- Structured timeline with key tasks for success in both R21 and R33 phase are described.

Weaknesses

- It is not evident how long and if local ethics approval will be required for each of the randomly selected health areas in the R33 phase.
- The R21 timeline does not describe plans for integrating findings in the three Aims to refine the eSkinHealth app prior to deployment in the R33 phase.

Protections for Human Subjects:

Unacceptable Risks and/or Inadequate Protections

- Children are included in the study but no discussion of how children would be consented.
- No discussion surrounding potential data breaches and use of photos to identify study participants. What happens if there are facial lesions or photos inadvertently include identifying information (e.g. facial features)?

Data and Safety Monitoring Plan (Applicable for Clinical Trials Only):

Unacceptable

- No data safety and monitoring plan described

Inclusion Plans:

- Sex/Gender: Distribution justified scientifically
- Race/Ethnicity: Distribution justified scientifically
- For NIH-Defined Phase III trials, Plans for valid design and analysis: Not applicable
- Inclusion/Exclusion Based on Age: Distribution justified scientifically

Vertebrate Animals:

Not Applicable (No Vertebrate Animals)

Biohazards:

Not Applicable (No Biohazards)

Applications from Foreign Organizations:

Justified

- Ulcerative skin disease is a significant problem in the Ivory Coast, and development of the proposed mHealth intervention will be of high value.
- Strong network of collaborators including leading national centers that diagnose and treat ulcerative skin disease suggests a high likelihood of success.

Select Agents:

Not Applicable (No Select Agents)

Resource Sharing Plans:

Unacceptable

- No resource sharing plan described in proposal.

Authentication of Key Biological and/or Chemical Resources:
Not Applicable (No Relevant Resources)

Budget and Period of Support:
Recommend as requested

THE FOLLOWING SECTIONS WERE PREPARED BY THE SCIENTIFIC REVIEW OFFICER TO SUMMARIZE THE OUTCOME OF DISCUSSIONS OF THE REVIEW COMMITTEE, OR REVIEWERS' WRITTEN CRITIQUES, ON THE FOLLOWING ISSUES:

PROTECTION OF HUMAN SUBJECTS: UNACCEPTABLE Reviewer 3 notes that children are included in the study but there is no discussion as how children would be consented. In addition, there is no consideration of potential data breaches and use of photos to identify study participants. What happens if there are facial lesions or photos inadvertently include identifying information?

INCLUSION OF WOMEN PLAN: ACCEPTABLE
INCLUSION OF MINORITIES PLAN: ACCEPTABLE
INCLUSION ACROSS THE LIFESPAN PLAN: ACCEPTABLE

SCIENTIFIC REVIEW OFFICER'S NOTE: The reviewers noticed that the applicants did not include the research resource sharing plans in their application. NIH considers the sharing of unique research resources developed through NIH-sponsored research an important means to enhance the value and further the advancement of research. When resources have been developed with NIH funds and the associated research findings published or provided to NIH, it is important that the results be made readily available for research purposes to qualified individuals within the scientific community.

COMMITTEE BUDGET RECOMMENDATIONS: The budget was recommended as requested.

Footnotes for 1 R21 TW011860-01; PI Name: Yotsu, Rie

Ad hoc or special section application percentiled against "Total CSR" base.

NIH has modified its policy regarding the receipt of resubmissions (amended applications).See Guide Notice NOT-OD-18-197 at https://grants.nih.gov/grants/guide/notice-files/NOT-OD-18-197.html. The impact/priority score is calculated after discussion of an application by averaging the overall scores (1-9) given by all voting reviewers on the committee and multiplying by 10. The criterion scores are submitted prior to the meeting by the individual reviewers assigned to an application, and are not discussed specifically at the review meeting or calculated into the overall impact score. Some applications also receive a percentile ranking. For details on the review process, see http://grants.nih.gov/grants/peer_review_process.htm#scoring.

MEETING ROSTER

Center for Scientific Review Special Emphasis Panel
CENTER FOR SCIENTIFIC REVIEW
PAR-19-376:Mobile Health: Technology and Outcomes in Low and Middle Income Countries
ZRG1 IMST-U (55)
03/24/2021 - 03/26/2021

Notice of NIH Policy to All Applicants: Meeting rosters are provided for information purposes only. Applicant investigators and institutional officials must not communicate directly with study section members about an application before or after the review. Failure to observe this policy will create a serious breach of integrity in the peer review process, and may lead to actions outlined in NOT-OD-14-073 at https://grants.nih.gov/grants/guide/notice-files/NOT-OD-14-073.html and NOT-OD-15-106 at https://grants.nih.gov/grants/guide/notice-files/NOT-OD-15-106.html, including removal of the application from immediate review.

CHAIRPERSON(S)

HIGHTOW-WEIDMAN, LISA B, MD, MPH
PROFESSOR
INSTITUTE FOR GLOBAL HEALTH
AND INFECTIOUS DISEASES
GILLINGS SCHOOL OF PUBLIC HEALTH
UNIVERSITY OF NORTH CAROLINA
CHAPEL HILL, NC 27599-7030

MEMBERS

ADAMS, ZACHARY WILLIAM, PHD
ASSISTANT PROFESSOR
DEPARTMENT OF PSYCHIATRY
INDIANA UNIVERSITY SCHOOL OF MEDICINE
INDIANAPOLIS, IN 46202

ARONSON, IAN DAVID, PHD
PRINCIPAL INVESTIGATOR
CENTER FOR TECHNOLOGY AND BEHAVIORAL HEALTH
NATIONAL DEVELOPMENT
AND RESEARCH INSTITUTES, INC
NEW YORK, NY 10010

BACHANI, ABDULGAFOOR M, PHD
ASSOCIATE PROFESSOR
INTERNATIONAL HEALTH
HEALTH SYSTEMS
JOHNS HOPKINS UNIVERSITY
BALTIMORE, MD 21205

BLINDER, VICTORIA S, MD
MEDICAL ONCOLOGIST
DIVISION OF EPIDEMIOLOGY AND BIOSTATISTICS
DEPARTMENT OF HEALTHCARE POLICY
MEMORIAL SLOAN KETTERING CANCER CENTER
NEW YORK, NY 10017

CAMPBELL, JOHN PETER, MPH, MD
ASSOCIATE PROFESSOR
DEPARTMENT OF OPHTHALMOLOGY
OREGON HEALTH AND SCIENCE UNIVERSITY
PORTLAND, OR 97239

CHAI, PETER R, MD
ASSISTANT PROFESSOR
DEPARTMENT OF EMERGENCY MEDICINE
BRIGHAM AND WOMEN'S HOSPITAL
BOSTON, MA 02115

CHU, KAR-HAI, PHD
ASSOCIATE PROFESSOR
DEPARTMENT OF BEHAVIORAL AND COMMUNITY
HEALTH SERVICES
GRADUATE SCHOOL OF PUBLIC HEALTH
UNIVERSITY OF PITTSBURGH
PITTSBURGH, PA 15261

CREMER, MIRIAM, MD, MPH
ASSOCIATE PROFESSOR
DEPARTMENT OF OBSTETRICS AND GYNECOLOGY
CLEVELAND CLINIC
CLEVELAND, OH 44195

DAHNE, JENNIFER RENEE, PHD
ASSISTANT PROFESSOR
DEPARTMENT OF PSYCHIATRY
MEDICAL UNIVERSITY OF SOUTH CAROLINA
CHARLESTON, SC 29425

DAVIS, JOHN LUCIAN, MD
ASSOCIATE PROFESSOR
DEPARTMENT OF EPIDEMIOLOGY AND MEDICINE
SCHOOL OF PUBLIC HEALTH
YALE UNIVERSITY
NEW HAVEN, CT 06520

ENAH, COMFORT CHU, MSN, PHD, RN
CHAIR AND ASSOCIATE PROFESSOR
DEPARTMENT OF HEALTH AND SOCIAL SCIENCES
SOLOMONT SCHOOL OF NURSING
UNIVERSITY OF MASSACHUSETTS LOWELL
LOWELL, MA 01854

FARQUHAR, CAREY, MD
PROFESSOR
DEPARTMENT OF GLOBAL HEALTH
SCHOOL OF PUBLIC HEALTH
UNIVERSITY OF WASHINGTON
SEATTLE, WA 98104

FRASER, HAMISH SF, MBBS, MS
ASSOCIATE PROFESSOR
DEPARTMENT OF BIOMEDICAL INFORMATICS
BROWN UNIVERSITY
PROVIDENCE, RI 02912

GIFFORD, ALLEN L, MD
PROFESSOR
DEPARTMENT OF PUBLIC HEALTH AND MEDICINE
BOSTON UNIVERSITY SCHOOL OF PUBLIC HEALTH
BOSTON, MA 02118

HAAS, NIINA M, MA
DIRECTOR OF RESEARCH OPERATIONS
BRIGHT OUTCOME INC.
BUFFALO GROVE, IL 60089-1998

HECKMAN, BRYAN WAYNE, PHD
ASSOCIATE PROFESSOR
DEPARTMENT OF PSYCHIATRY
AND BEHAVIORAL SCIENCES
MEHARRY MEDICAL COLLEGE
NASHVILLE, TN 37208

HELLERINGER, STEPHANE, PHD
PROFESSOR
SOCIAL SCIENCE DIVISION
SOCIAL RESEARCH AND PUBLIC POLICY PROGRAM
NEW YORK UNIVERSITY ABU DHABI
ABU DHABI
UNITED ARAB EM

HENDERSON, KIERSTEN, PHD
CHIEF EXECUTIVE OFFICER
PARASITE ID, CORP.
SEATTLE, WA 98117

ICARD, LARRY D, DSW, PHD
PROFESSOR AND DIRECTOR
CENTER ON INTERVENTION AND PRACTICE RESEARCH
SCHOOL OF SOCIAL WORK
TEMPLE UNIVERSITY
PHILADELPHIA, PA 19122

IWELUNMOR, JULIET, PHD
ASSOCIATE PROFESSOR
DEPARTMENT OF BEHAVIORAL SCIENCE AND
HEATLH EDUCATION
COLLEGE FOR PUBLIC HEALTH AND SOCIAL JUSTICE
SAINT LOUIS UNIVERSITY
ST. LOUIS, MO 63104

JACOBSON, JUDITH S, MPH, DRPH
ASSOCIATE PROFESSOR
DEPARTMENT OF EPIDEMIOLOGY
MAILMAN SCHOOL OF PUBLIC HEALTH
COLUMBIA UNIVERSITY
NEW YORK, NY 10032

JONES, DEBORAH LYNNE, PHD
PROFESSOR
DEPARTMENT OF PSYCHIATRY
AND BEHAVIORAL SCIENCES
MILLER SCHOOL OF MEDICINE
UNIVERSITY OF MIAMI
MIAMI, FL 33136

KUHNS, LISA MARY, MPH, PHD
ASSOCIATE PROFESSOR
DEPARTMENT OF PEDIATRICS
FEINBERG SCHOOL OF MEDICINE
NORTHWESTERN UNIVERSITY
CHICAGO, IL 60612

LEE, HAEOK, DNSC
PROFESSOR
DEPARTMENT OF NURSING
UNIVERSITY OF MASSACHUSETTS BOSTON
BOSTON, MA 02125

LEE, HEE YUN, PHD
PROFESSOR
DEPARTMENT OF SOCIAL WORK
THE UNIVERSITY OF ALABAMA AT TUSCALOOSA
TUSCALOOSA, AL 35487

LEHMAN, WAYNE E.K, PHD
SENIOR RESEARCH SCIENTIST
INSTITUTE OF BEHAVIORAL RESEARCH
TEXAS CHRISTIAN UNIVERSITY
FORT WORTH, TX 76129

LIU, LONGJIAN, MD, PHD
ASSOCIATE PROFESSOR
DEPARTMENT OF EPIDEMIOLOGY AND BIOSTATISTICS
DREXEL UNIVERSITY
PHILADELPHIA, PA 19104

MCGINNIS, RYAN S, PHD
ASSISTANT DIRECTOR
DEPARTMENT OF ELECTRICAL AND BIOMEDICAL
ENGINEERING
THE UNIVERSITY OF VERMONT
BURLINGTON, VT 05405

MODAVE, FRANCOIS PAUL PHILIPPE, PHD
ASSOCIATE PROFESSOR
DEPARTMENT OF HEALTH OUTCOMES
AND BIOMEDICAL INFORMATICS
COLLEGE OF MEDICINE, UNIVERSITY OF FLORIDA
GAINESVILLE, FL 32601

MOORE, DAVID J, PHD
ASSOCIATE PROFESSOR
DEPARTMENT OF PSYCHIATRY
UNIVERSITY OF CALIFORNIA-SAN DIEGO
SAN DIEGO, CA 92103

NGO, VICTORIA KHANH, PHD
BEHAVIORAL AND SOCIAL SCIENTIST
DEPARTMENT OF COMMUNITY HEALTH
AND SOCIAL SCIENCES
CITY UNIVERSITY OF NEW YORK
NEW YORK CITY, NY 10027

OSTERMANN, JAN, PHD
ASSOCIATE PROFESSOR
DEPARTMENT OF HEALTH SERVICES POLICY
AND MANAGEMENT
ARNOLD SCHOOL OF PUBLIC HEALTH
UNIVERSITY OF SOUTH CAROLINA
COLUMBIA, SC 29208

PATEL, VIMLA L, DSC, PHD
SENIOR RESEARCH SCIENTIST AND DIRECTOR
CENTER FOR COGNITIVE STUDIES IN MEDICINE
AND PUBLIC HEALTH
THE NEW YORK ACADEMY OF MEDICINE
NEW YORK, NY 10029

REYNOLDS, NANCY R, PHD
PROFESSOR
DEPARTMENT OF COMMUNITY AND PUBLIC HEALTH
JOHNS HOPKINS SCHOOL OF NURSING
BALTIMORE, MD 21205

ROMSKI, MARYANN, PHD
REGENTS PROFESSOR
DEPARTMENTS OF COMMUNICATION, PSYCHOLOGY,
AND COMMUNICATION SCIENCES AND DISORDERS
GEORGIA STATE UNIVERSITY
ATLANTA, GA 30302

SEKANDI, JULIET NABBUYE, MD, DRPH
ASSISTANT PROFESSOR
COLLEGE OF PUBLIC HEALTH
UNIVERSITY OF GEORGIA
ATHENS, GA 30602

TAYO, BAMIDELE OLUSEGUN, PHD
ASSOCIATE PROFESSOR
DEPARTMENT OF PUBLIC HEALTH SCIENCES
STRITCH SCHOOL OF MEDICINE
LOYOLA UNIVERSITY OF CHICAGO
MAYWOOD, IL 60660

TORIOLA, ADETUNJI T, MD, MPH, PHD
ASSISTANT PROFESSOR
DEPARTMENT OF SURGERY
DIVISION OF PUBLIC HEALTH SERVICES
WASHINGTON UNIVERSITY SCHOOL OF MEDICINE
ST LOUIS, MO 63110

TUCKER, JOAN S, PHD
SENIOR BEHAVIORAL SCIENTIST
RAND HEALTH
RAND CORPORATION
SANTA MONICA, CA 90401

VORDERSTRASSE, ALLISON AMEND, DNSC, MSN
PROFESSOR AND DEAN
SCHOOL OF NURSING
UNIVERSITY OF MASSACHUSETTS AMHERST
AMHERST, MA 01003

WANG, BING, PHD
ASSOCIATE PROFESSOR
DEPARTMENT OF COMPUTER SCIENCE AND ENGINEERING
SCHOOL OF ENGINEERING
UNIVERSITY OF CONNECTICUT
STORRS, CT 06269

WOLFF, MARK STEVEN, DDS, PHD
PROFESSOR
DIVISION OF RESTORATIVE DENTISTRY
SCHOOL OF DENTAL MEDICINE
UNIVERSITY OF PENNSYLVANIA
PHILADELPHIA, PA 19104

WYCHE, SUSAN, PHD
ASSOCIATE PROFESSOR
DEPARTMENT OF MEDIA AND INFORMATION
MICHIGAN STATE UNIVERSITY
EAST LANSING, MI 48823

YAWN, BARBARA P, MD
CHIEF CLINICAL OFFICER, COPD FOUNDATION
ADJUNCT PROFESSOR
DEPARTMENT OF FAMILY AND COMMUNITY HEALTH
UNIVERSITY OF MINNESOTA
MINNEAPOLIS, MN 55449

ZANONI, BRIAN C, MD, MPH
ASSISTANT PROFESSOR
DEPARTMENT OF PEDIATRICS
EMORY UNIVERSITY SCHOOL OF MEDICINE
ATLANTA, GA 30322

MAIL REVIEWER(S)

STATON, CATHERINE ANN, MD
ASSOCIATE PROFESSOR
DEPARTMENT OF SURGERY
DUKE UNIVERSITY
DURHAM, NC 27701

SCIENTIFIC REVIEW OFFICER

KRISHNARAJU, RAJ K, PHD
SCIENTIFIC REVIEW OFFICER
CENTER FOR SCIENTIFIC REVIEW
NATIONAL INSTITUTES OF HEALTH
BETHESDA, MD 20892

EXTRAMURAL SUPPORT ASSISTANT

CAMPITELLI, CHRISTINA MARIE
EXTRAMURAL SUPPORT ASSISTANT (INTERN)
CENTER FOR SCIENTIFIC REVIEW
BETHESDA, MD 20747

Consultants are required to absent themselves from the room during the review of any application if their presence would constitute or appear to constitute a conflict of interest.
